# Manipulating the Bacterial Cell Cycle and Cell Size by Titrating the Expression of Ribonucleotide Reductase

**DOI:** 10.1128/mBio.01741-17

**Published:** 2017-11-14

**Authors:** Manlu Zhu, Xiongfeng Dai, Weilun Guo, Zengxiang Ge, Mingxuan Yang, Haikuan Wang, Yi-Ping Wang

**Affiliations:** a State Key Laboratory of Protein and Plant Gene Research, School of Life Sciences, Peking University, Beijing, China; b School of Life Sciences, Central China Normal University, Wuhan, China; University of Rochester

**Keywords:** C period, cell size, ribonucleotide reductase, cell cycle, dNTP

## Abstract

Understanding how bacteria coordinate growth with cell cycle events to maintain cell size homeostasis remains a grand challenge in biology. The period of chromosome replication (C period) is a key stage in the bacterial cell cycle. However, the mechanism of *in vivo* regulation of the C period remains unclear. In this study, we found that titration of the expression of ribonucleotide reductase (RNR), which changes the intracellular deoxynucleoside triphosphate (dNTP) pools, enables significant perturbations of the C period, leading to a substantial change in cell size and DNA content. Our work demonstrates that the intracellular dNTP pool is indeed an important parameter that controls the progression of chromosome replication. Specially, RNR overexpression leads to a shortened C period compared with that of a wild-type strain growing under different nutrient conditions, indicating that the dNTP substrate levels are subsaturated under physiological conditions. In addition, perturbing the C period does not significantly change the D period, indicating that these two processes are largely independent from each other. Overall, titration of ribonucleotide reductase expression can serve as a standard model system for studying the coordination between chromosome replication, cell division, and cell size.

## IMPORTANCE

Bacteria must coordinate growth with cell cycle progression to maintain cell size hemostasis. Cell cycle and cell size regulation is a fundamental concern in biology. The period required for chromosome replication (the C period) is a key stage in the bacterial cell cycle. However, how the C period is controlled *in vivo* remains largely an open question in this field of bacterial cell cycle regulation. Through introducing a genetic circuit into *Escherichia coli* for titrating the expression of ribonucleotide reductase, we achieve substantial perturbation of the C period and cell size. Our work demonstrates that the intracellular dNTP pool is an important parameter that controls the progression of chromosome replication. Moreover, our work indicates that bacterial cells manage to maintain subsaturated dNTP levels under different nutrient conditions, leading to a submaximal speed of DNA replication fork movement.

## OBSERVATION

The tight coordination between biomass growth and cell cycle events, including chromosome replication and cell division, to maintain cell size homeostasis is a fundamental feature of various types of prokaryotic and eukaryotic cells (1–7). As proposed in the Cooper and Helmstetter model, the C period (the period required for chromosome replication) and the D period (the period between the end of replication and the completion of division) are the two key stages in the bacterial cell cycle (8, 9). Recent quantitative studies have demonstrated that the bacterial cell size is closely related to growth rate and cell cycle progression (C plus D periods) with various growth perturbations (10–12).

Although cell cycle progression is closely related to cell size, much less is known about how bacterial cells manage to control the length of time of their cell cycle. The D period can be perturbed at the step of septum formation by means of division machinery, such as FtsZ (12–14). The C period can be changed by thymine limitation, which is imposed extracellularly (15, 16), or by mutations of replisome proteins (17, 18). However, how the C period is controlled *in vivo* remains largely unclear. Here we show that titration of the ribonucleotide reductase (RNR) expression level, which changes the intracellular deoxynucleoside triphosphate (dNTP) pools, causes significant perturbation of the C period and of bacterial cell size.

**FIG 1:**
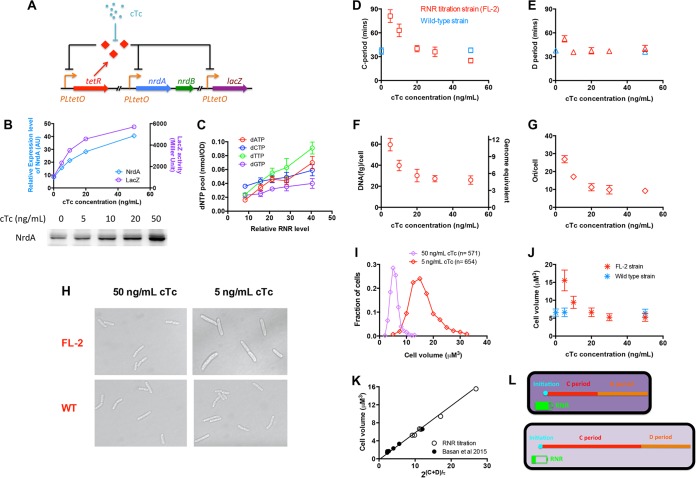
Manipulating the bacterial cell cycle and cell size by titrating the expression of ribonucleotide reductase. (A) Key construct in the chromosome of RNR titration strain (FL-2 strain). (B) Relative levels of expression of NrdA and LacZ of the titration strain under various concentrations of the cTc inducer. The RNR titration strain in this study was always grown in LB medium. AU, arbitrary units. (C) Correlation between the dNTP pools and the level of expression of RNR. (D) C periods of the RNR titration strain (red) and wild-type cells (blue) under different concentrations of cTc. Data points are the averages of results from triplicate experiments. Error bars denote standard deviations. (E) D period of the RNR titration strain (red) and wild-type strain (blue) under different concentrations of cTc. (F) DNA content per cell under various concentrations of cTc. The right *y* axis shows the genome equivalent per cell. Data points are the averages of results from triplicate experiments. Error bars denote standard deviations. (G) Numbers of the replication origins (Ori) per cell under various concentrations of cTc. (H) Cell images of the RNR titration strain and wild-type strain (WT) with 50 ng/ml cTc and 5 ng/ml cTc. (I) Distributions of cell volumes for the RNR titration strain with 50 ng/ml cTc and 5 ng/ml cTc. (J) Cell volume of the RNR titration strain and wild-type strain under different concentrations of cTc. Data points are the averages of results with 500 to 1,000 individual cells. (K) Linear correlation between cell size and 2^(C + D)/τ^ upon RNR titration together with nutrient limitation (Table S1). (L) Schematic representation of the effect of RNR titration on cell cycle and cell size. When the RNR level is high (upper cell), the intracellular dNTP supply is high (denoted with the dark-purple background), leading to a higher speed of replication fork movement and a shorter C period. In contrast, a low RNR level (lower cell) causes a reduction in dNTPs (denoted with the light-purple background), leading to a remarkably longer C period and a delayed cell cycle. Therefore, the delayed cell cycle (C + D period) causes a significantly increased cell volume.

The C period reflects the moving speed of the replication fork during DNA replication. Since DNA replication can be considered an enzymatic process catalyzed by DNA polymerase, we supposed that perturbing the dNTP substrate pools might effectively achieve the perturbation of the C period. RNR catalyzes the reduction of ribonucleoside diphosphate to deoxyribonucleoside diphosphate, which is the rate-limiting step in dNTP production (19–21). In *Escherichia coli*, the class Ia RNR (encoded by the *nrdA* and *nrdB* genes) is responsible for dNTP production under aerobic conditions (22). Thus, we introduced a genetic circuit into a wild-type *E. coli* K-12 strain in order to titrate the expression of RNR (FL-2 strain). For this purpose, we replaced the original promoter of the *nrdAB* operon (containing *nrdA* and *nrdB*) in the *E. coli* chromosome with a strong P_*LtetO*_ promoter and introduced a P_*LtetO*_-*tetR* cassette into the chromosome (Fig. 1A). In this case, the expression of the *nrd* operon was controlled by an auto-negative-feedback loop. The *lacZ* gene in the chromosome was also under the control of the same P_*LtetO*_ promoter so that LacZ could be taken as the reporter for conveniently monitoring the relative expression activity of the P_*LtetO*_ promoter. By adjusting the concentration of the TetR inducer chlortetracycline (cTc), we were able to quantitatively titrate the expression level of the RNR (measured by Western blotting assay and LacZ reporter activity) (Fig. 1B). We then started to characterize related parameters of the RNR titration strain growing in LB medium. As a starting point, we first measured the dNTP pools upon change of RNR levels by high-performance liquid chromatography (HPLC). All of the four dNTP pools were indeed perturbed, correlating well the RNR levels (Fig. 1C). Especially, the pools of dATP and dTTP had been changed by 4- to 5-fold. However, the changes in dCTP and dGTP levels were mild. Although dNTP pools changed significantly upon RNR titration, the ribonucleoside triphosphate (rNTP) pools remained constant (see Fig. S1 in the supplemental material).

10.1128/mBio.01741-17.2 FIG S1  rNTP pools upon RNR titration. The relative RNR level corresponds to the value shown in Fig. 1C. Download FIG S1, PDF file, 0.02 MB. Copyright © 2017 Zhu et al. 2017 Zhu et al. This content is distributed under the terms of the Creative Commons Attribution 4.0 International license.

We then characterized the cell cycle parameters and DNA content of the RNR titration strain. The C period was measured by both quantitative PCR (qPCR) and the DNA increment method (see below). Strikingly, the C period strongly changed upon RNR titration. As shown in Fig. 1D, the C period increased over 3-fold (from 25 min to 81 min) with reduced RNR levels (from 50 ng/ml cTc to 5 ng/ml cTc). Moreover, the overexpression of RNR (50 ng/ml cTc) might even cause a remarkably shorter C period (25 min) than that of wild-type cells (38 min) (Fig. 1D). Given that the growth rate remained largely unchanged in the cTc range studied (Fig. S2), RNR titration enabled the decoupling of the C period and the growth rate. Although causing substantial change of the C period, the RNR titration had no significant effect on the D period (calculated from the C period and number of origins of replication per cell) (Fig. 1E). Moreover, we found that cellular DNA content and replication origin (per cell quantity) increased up to 3-fold with the reduced RNR level (Fig. 1F and G).

10.1128/mBio.01741-17.3 FIG S2  Doubling times of the RNR titration strain under various concentrations of cTc. Download FIG S2, PDF file, 0.02 MB. Copyright © 2017 Zhu et al. 2017 Zhu et al. This content is distributed under the terms of the Creative Commons Attribution 4.0 International license.

We further investigated whether the altered C period could cause the change in cell size of the RNR titration strain. The cell size was directly determined by microscopy and ImageJ software analysis, with further verification from measuring the optical density at 600 nm (OD_600_) per cell (Fig. S3). Cell size increased remarkably with the prolonged C period at low levels of expression of RNR (Fig. 1H, I, and J). From 50 ng cTc/ml (C period, 25 min) to 5 ng/ml cTc (C period, 81 min), cell size was enlarged by over 3-fold. In this case, the change in cell size correlated well with the change in cellular DNA content (Fig. 1F and Fig. S4). The increase in cell size was achieved by a dramatic increase in length and a slight increase in width (Fig. S5).

10.1128/mBio.01741-17.4 FIG S3  Correlation of OD/10^9^ cells versus cell size for the RNR titration strain. The OD per cell (average mass per cell) correlates well with the cell volume obtained from imaging. Download FIG S3, PDF file, 0.02 MB. Copyright © 2017 Zhu et al. 2017 Zhu et al. This content is distributed under the terms of the Creative Commons Attribution 4.0 International license.

10.1128/mBio.01741-17.5 FIG S4  Correlation between cellular DNA content and cell size. Data plotted here include the results with the RNR titration strain (Fig. 1F and J) and previous results of Basan et al. (Basan M, et al., Mol Syst Biol 11:836, 2015). Download FIG S4, PDF file, 0.03 MB. Copyright © 2017 Zhu et al. 2017 Zhu et al. This content is distributed under the terms of the Creative Commons Attribution 4.0 International license.

10.1128/mBio.01741-17.6 FIG S5  Cell length and cell width of the RNR titration strain. (A) Average cell lengths of the RNR titration strain and wild-type strain under different concentrations of cTc; (B) average cell widths of the RNR titration strain and wild-type strain under different concentrations of cTc. Error bars denote standard deviations. Data points are the averages of results with 500 to 1,000 individual cells. Download FIG S5, PDF file, 0.03 MB. Copyright © 2017 Zhu et al. 2017 Zhu et al. This content is distributed under the terms of the Creative Commons Attribution 4.0 International license.

The relation between cell size, cell cycle progression (C + D), and growth rate can be quantitatively described by the following equation:

(1) $$V=V_{i}\times 2^{(\text{C }+\text{ D})/\tau }$$

where *V* is the average cell volume, *V*_*i*_ is the initiation volume per chromosome origin (referred to as “initiation mass” or “unit cell”) (10), and τ is the mass doubling time. This relation was originally proposed by Donachie in 1968 to explain the positive correlation between cell size and growth rate under different nutrient conditions in which C + D remains almost constant (23). Recent studies have generalized the applicability of equation 1 with various modes of growth perturbations in which growth rate and cell cycle can be extensively perturbed (10, 12). The initiation mass was found to be constant with various growth limitations (10). We plotted the cell size versus the scaling factor, 2^(C + D)/τ^, for the RNR titration strain (open black circles in Fig. 1K; see also Table S1 in the supplemental material) together with that for wild-type cells under nutrient limitation (solid black circles in Fig. 1K; Table S1). Cell size was indeed linearly proportional to the scaling factor, 2^(C + D)/τ^, demonstrating that the initiation mass is still constant even in the range of a 10-fold change in cell size.

10.1128/mBio.01741-17.7 TABLE S1  Cell size, DNA content, and cell cycle parameters for the RNR titration strain and wild-type NCM3722 strain under nutrient limitation. Download TABLE S1, DOCX file, 0.02 MB. Copyright © 2017 Zhu et al. 2017 Zhu et al. This content is distributed under the terms of the Creative Commons Attribution 4.0 International license.

**FIG 2:**
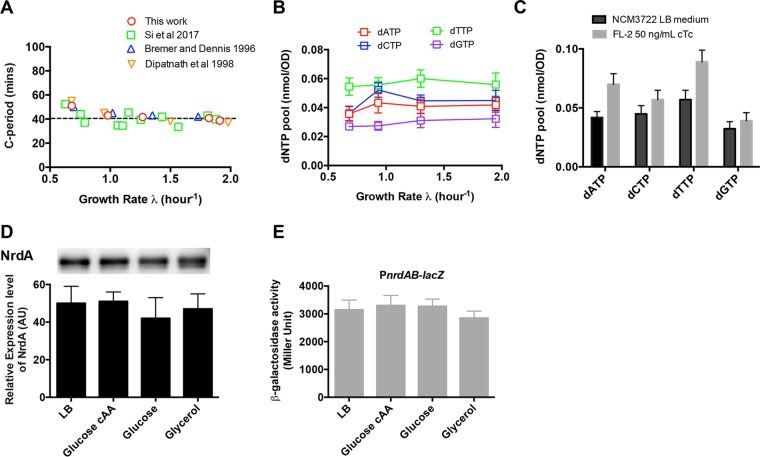
C period, dNTP pools, and RNR expression level under different nutrient conditions. (A) C period of the *E. coli* wild-type K-12 NCM3722 strain under different nutrient conditions. The dashed line denote a constant 40 min. (B) dNTP pools of wild-type *E. coli* under four nutrient conditions, namely, LB medium (λ, 1.9/h), glucose plus Casamino Acids (cAA) medium (λ, 1.3/h), glucose medium (λ, 0.97/h), and glycerol medium (λ, 0.69/h). (C) Comparison of the dNTP pools between the wild-type strain growing in LB medium (C period, 38 min) and the RNR titration strain growing in LB medium plus 50 ng/ml cTc (C period, 25 min). (D) Relative RNR expression levels (the intensity of the NrdA bands was resolved by Western blotting) under four nutrient conditions. (E) The relative RNR expression level (determined by the P_*nrdAB*_-*lacZ* reporter with the FL-3 strain) under four nutrient conditions.

Our study on RNR titration strongly suggests that the dNTP pool (limited by RNR level) is indeed an important molecular factor that limits the C period. The dNTP pools can also be downregulated by thymine limitation (solely changes dTTP) (16) and hydroxyurea treatment (10), both of which are imposed extracellularly. Instead, we achieved systematic and robust perturbations of all four dNTPs through titrating the RNR expression *in vivo*, allowing both downregulation and upregulation of dNTP pools. An interesting finding is that RNR overexpression even leads to a significantly smaller C period (25 min) than the value reported under good nutrient conditions (remains constant at ~40 min) (Fig. 2A) (10, 24, 25). This corresponds to the shortest C period ever reported for *E. coli*, suggesting that the dNTP substrates and the speed of replication fork movement are always subsaturated under different nutrient conditions. Direct measurement indeed shows that the dNTP pools remained constant under four nutrient conditions (Fig. 2B) and were significantly lower than the value upon RNR overexpression (Fig. 2C). With Western blotting and a *lacZ* reporter assay, we further demonstrated that the expression level of RNR also remained largely constant under four nutrient conditions (Fig. 2D and E). Those results indicate that *E. coli* tightly maintains the hemostasis of dNTP pools through keeping a constant RNR level under these conditions. This might be crucial for *E. coli* growing under physiological conditions, as significantly increased dNTP pools are likely to stimulate mutagenesis (26, 27), thus compromising replication fidelity.

It would be interesting to know whether the C period is limited mainly by one specific dNTP or by all of them. In the case of RNR titration, both dATP and dTTP levels change by 4- to 5-fold, while dGTP and dCTP levels change mildly (1.5-fold). This indicates that it is dATP and dTTP that mainly drive the C-period perturbations upon RNR titration. This is further supported by the fact that dATP and dTTP significantly increase while dGTP and dCTP increase only marginally when the C period decreases from 38 min to 25 min (Fig. 2C).

It has long been proposed that the D period runs after the C period (9). However, it is unclear whether the C period interferes with the D period. Our quantitative study shows that perturbing the C period does not significantly change the D period, thus clarifying that these two processes are largely independent of each other. Overall, our study shows that RNR titration is a solid molecular way for significantly perturbing the cell cycle and cell size (see the schematic representation in Fig. 1L) and can also serve as a standard model system for studying the coordination between chromosome replication, cell division, and cell size.

10.1128/mBio.01741-17.1 TEXT S1  Supplemental methods. Download TEXT S1, DOCX file, 0.05 MB. Copyright © 2017 Zhu et al. 2017 Zhu et al. This content is distributed under the terms of the Creative Commons Attribution 4.0 International license.
