# Supplementary figures and images for: Manipulating the Bacterial Cell Cycle and Cell Size by Titrating the Expression of Ribonucleotide Reductase
Source: mBio. 2017 Nov 14;8(6):e01741-17. doi: 10.1128/mBio.01741-17 (PMC5686538; doi:10.1128/mBio.01741-17)

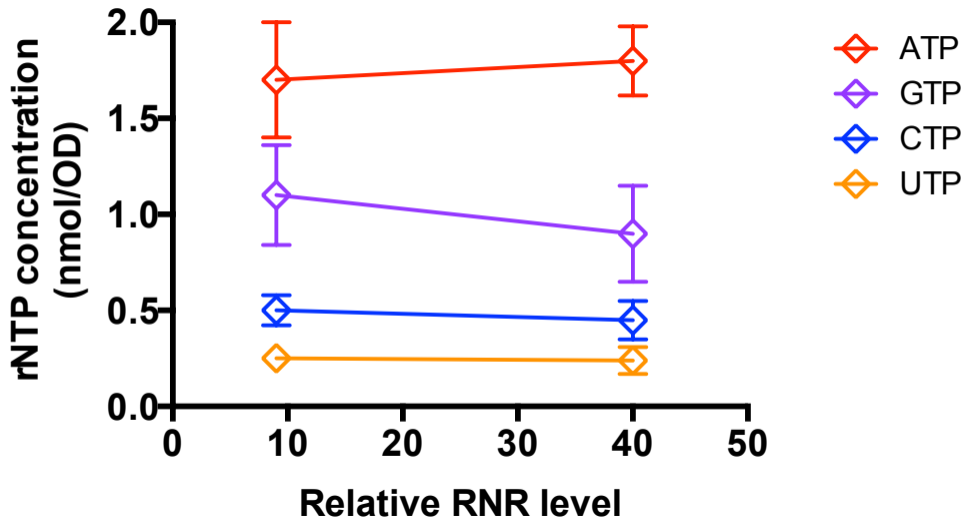

Supplement: FIG S1 [file mbo006173599sf1.pdf]

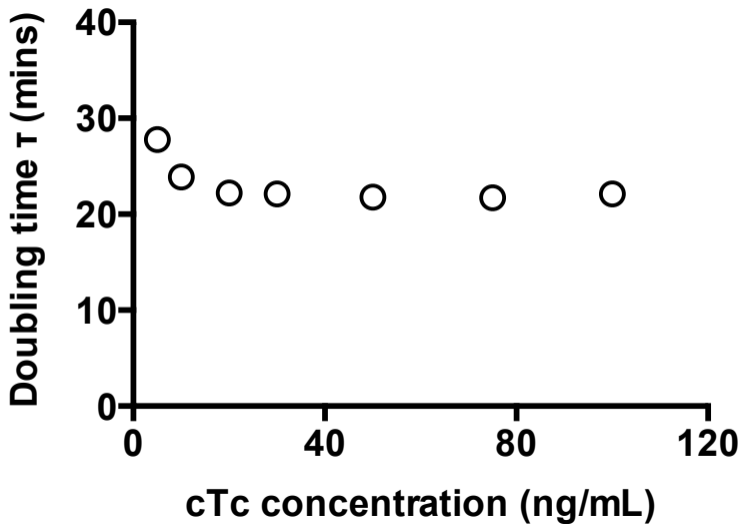

Supplement: FIG S2 [file mbo006173599sf2.pdf]

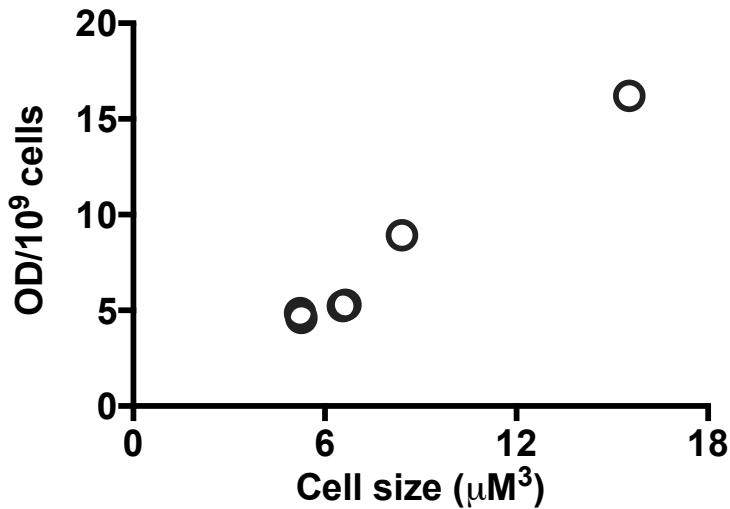

Supplement: FIG S3 [file mbo006173599sf3.pdf]

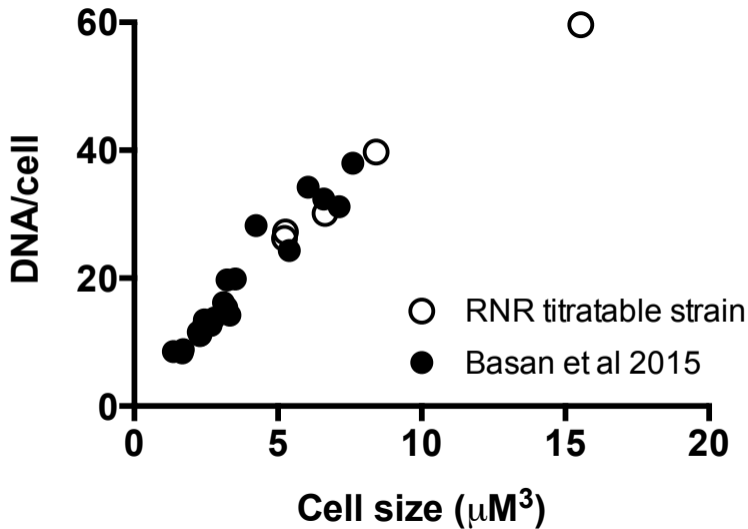

Supplement: FIG S4 [file mbo006173599sf4.pdf]

**A**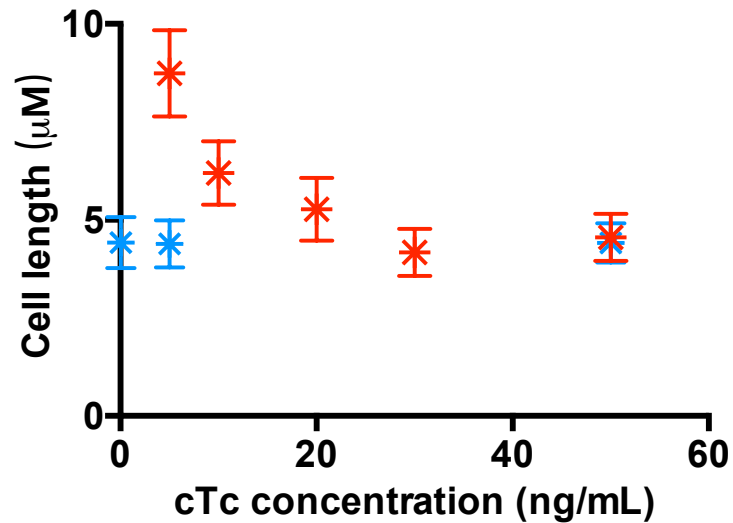**B**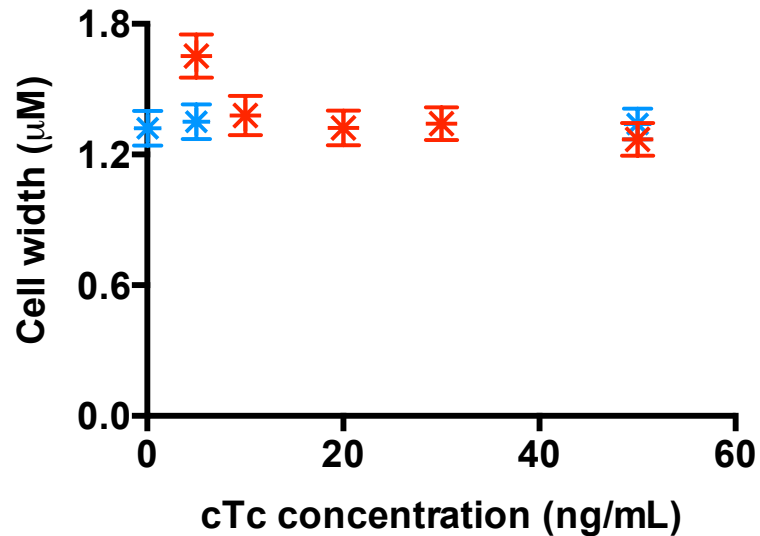

Supplement: FIG S5 [file mbo006173599sf5.pdf]
