# Supplementary material for: Manipulating the Bacterial Cell Cycle and Cell Size by Titrating the Expression of Ribonucleotide Reductase
Source: mBio. 2017 Nov 14;8(6):e01741-17. doi: 10.1128/mBio.01741-17 (PMC5686538; doi:10.1128/mBio.01741-17)
Supplement: TABLE S1 [file mbo006173599st1.docx]

| Strain | Conditions | Genome equivalents/cell^a^ | Ori/genome^b^ | Ori/cell^c^ | C Period (mins) | D Period  (min) ^d^ | τ  (min) | Cell size (μM^3^) |
| --- | --- | --- | --- | --- | --- | --- | --- | --- |
| RNR titration strain | LB 5 ng/mL cTc | 12.68±1.4 | 2.12±0.05 | 26.89 | 81±7 | 52 | 28.1±0.4 | 15.5 |
|  | LB 10 ng/mL cTc | 8.45±0.9 | 2.02±0.06 | 17.07 | 63±6 | 36 | 24.1±0.3 | 9.4 |
|  | LB 20 ng/mL cTc | 6.41±0.5 | 1.75±0.04 | 11.23 | 40±4 | 37 | 22.2±0.2 | 6.6 |
|  | LB 30 ng/mL cTc | 5.79±0.4 | 1.70±0.03 | 9.85 | 36±6 | 37 | 22.1±0.3 | 5.3 |
|  | LB 50 ng/mL cTc | 5.51±0.5 | 1.65±0.05 | 9.09 | 26±3 | 40 | 20.7±0.2 | 5.2 |
| Wild type NCM3722 | LB broth | 6.45±0.6 | 1.80±0.05 | 11.62 | 38±2 | 36 | 21.3±0.3 | 6.5 |
|  | RDM+glucose | 6.50 | 1.75±0.04 | 11.38 | 41±1 | 39.7 | 23.0 | 6.6 |
|  | Glucose cAA | 3.02 | 1.72±0.06 | 5.19 | 42±3 | 35.2 | 32.5 | 3.3 |
|  | Glucose | 2.34 | 1.70±0.06 | 3.81 | 44±3 | 37 | 42.0 | 2.3 |
|  | Glycerol | 1.87 | 1.45±0.05 | 2.71 | 48±4 | 38 | 60.0 | 1.7 |
|  | Acetate | 1.76 | 1.30±0.05 | 2.29 | 58±4 | 50 | 90.0 | 1.7 |
|  | Mannose | 1.81 | 1.25±0.03 | 2.27 | 68±6 | 48 | 98.0 | 1.4 |

**Table S1 Cell size, DNA content and cell cycle parameters for the RNR titration strain and wild type NCM3722 strain under nutrient limitation.** Data marked in Red are taken from Basan et al(7).

1. Genome equivalents/cell is obtained using DNA/cell to divide the molecular mass of *E. coli* chromosome.
2. Ori/genome equivalent is obtained from the DNA accumulation curve after block of chromosome replication initiation (Figure S2).
3. Ori/cell is obtained with the data of a. and b.
4. D period is calculated from the value of ori/cell, C period and τ (mass doubling time) based on the equation(9, 13): ori/cell=2^(C+D)/τ^.
