# Supplementary material for: Manipulating the Bacterial Cell Cycle and Cell Size by Titrating the Expression of Ribonucleotide Reductase
Source: mBio. 2017 Nov 14;8(6):e01741-17. doi: 10.1128/mBio.01741-17 (PMC5686538; doi:10.1128/mBio.01741-17)
Supplement: TEXT S1 [file mbo006173599s1.docx]

**Materials and Methods**

**Strain construction**

Strains used in the study included wild type *E. coli* NCM3722 strain(1) and its derivatives：FL-2 strain, which was the RNR titration strain, and FL-3 strain, used for measuring the expression level of P*nrdAB*-LacZ.

To construct the FL-2 strain, the whole *kmR*-rrnBT-P*LtetO* cassette was first PCR amplified from the genome of NQ122 strain (carrying *kmR*-rrnBT-P*LtetO*-LacZ cassette)(2). The cassette was further integrated into the chromosome of NQ122-1 strain (with the *kmR* gene in NQ122 strain being flipped out by pCP20) to replace the native P*nrdAB* promoter region including its upstream sequence (-700 to -1, relative to the translation start site of *nrdA* gene) through λ-Red recombination system(3). The YcaC/YcaD site of the genome of the resulting strain was further integrated into a P*LtetO*-*tetR* cassette through λ-Red recombination system. This leads to the RNR titration strain, FL-2.

To construct the FL-3 strain, the entire promoter region and upstream sequence of the *nrdAB* operon together with the first seven codons of the *nrdA* gene (-700 to +21, relative to the translational start site of *nrdA* gene) was PCR amplified and inserted into the *Hin*dIII/*Bam*HI site of the low copy RK2-derived vector, pGD926(4), to be fused in frame with the eighth codons of the *LacZ* gene. This results vector pGD-P*nrdAB*. The pGD-P*nrdAB* vector was then transferred to a *lacZ*-deficient derivative of NCM3722 (5) to obtain FL-3 strain.

**Measurement of dNTP pools by HPLC**

100 mL exponentially growing cells (OD_600_ ~0.4) were filtered, washed with 10 mL cold saline solution, and further extracted with 5 mL cold 70% methanol for 5 hours at -20 ºC. 10 nmol of dITP was added to all the samples as an internal standard. The cell extracts were lyophilized, suspended in 0.5 mL ddH_2_O and further extracted by 0.5 mL chloroform. The aqueous phase was collected, lyophilized again and finally suspended in 0.1 mL water. Nucleotides pools were analyzed with the reversed-phase HPLC using a C_18_ column (UV detection at 254 nm by the Agilent 1260 infinity HPLC system) as described similarly as Buckstein et al(6). The commercialized solutions of four dNTPs (Sigma) and four rNTPs (Sigma) were used as standard reagents during analysis.

**Measurement of β-galactosidase (LacZ) activity**

Determination of LacZ activity was performed the same as described in You et al(2) based on the Miller’s ONPG (O-nitrophenyl-β-D-galactopyranoside) colorimetric method.

**Western blot assay**

For the western blot assay of the NrdA protein in the RNR titration strain, cell cultures in LB medium (COOLABER, Beijing) supplemented with various concentrations of chlortetracycline (cTc) were grown exponentially in LB medium to OD_600_ ~ 0.3. Around 10 μg total protein was subject to 12.5% SDS-PAGE for 2 hours under a constant 150 V voltage. The protein was further electrophoretically transferred to PVDF membranes (Millipore, USA) under a constant 200 mA current. The PVDF membrane was then blocked overnight in phosphate-buffered saline supplemented with 0.1% (v/v) Tween-20 (PBST) and 5% (w/v) skim milk (Difco). The membrane was further incubated with primary anti-NrdA rabbit polyclonal antibody (1:3000) in PBST containing 5% (w/v) skim milk (Difco) for 2 hours at room temperature, followed by being washed five times (at a 5 mins interval) with PBST. The membrane was subsequently incubated with the secondary-goat anti-rabbit IgG antibodies (HRP conjugated, Biorbyt) (1:3000) in PBST containing 5% (w/v) skim milk (Difco) for 2 hours at room temperature, followed by being washed five times (at a 5 mins interval) with PBST again. HPR enzymatic reaction was performed with an ECL western blotting detection kit (Amersham) and further detected using a 4200 Chemiluminescence Analyzer (Tanon, China).

**Measurement of DNA content**

Total DNA quantification is based on the diphenylamine colorimetric method as the same described in Basan et al(7).

**Measurement of C-period, D period, DNA content and origins/cell**

Measurement of the C-period is based on two methods. The first method is based on measuring the *ori/ter* ratio by qPCR as described in Si et al(8) and Hill et al(9). For details: 2 mL bacteria culture (OD600~0.4) was taken and immediately frozen in liquid nitrogen before sending to freezer for storage. On the next day, the bacteria genome was extracted using a bacteria total genome DNA extraction kit (TIANGEN BIOTECH (BEIJING) CO., LTD.). The DNA concentration was quantified through measuring the absorbance at 260 nm UV light with the NanoDrop ND-1000 UV-Vis Spectrophotometer (Thermo Scientific). Primers (TSINGKE BIOTECH CO) used for amplifying the DNA region proximal to the origin (*oriC*) and terminus (*ter*) are as follows(10): 3923874fw (5’-GCCCTGTGGATAACAAGGAT-3’) and 3923874rv (5’-CCTCATTCTGATCC CAGCTT-3’) for *oriC*, ter-fw (5’-TCCTCGCTGTTTG TCATCTT) and ter-rv (5’-GG TCTTGCTCGAATCCCTT) for *ter*. The qPCR reactions were performed with a SuperReal Premix SYBR Green Plus kit (TIANGEN BIOTECH (BEIJING) CO., LTD.) according to the manual. For each PCR reaction, each 20 μl sample contained 10 ng of DNA, 0.6 pmol of each primer and 10 μl of 2x SYBR Green Supermix. The reaction process was carried out in an Opticon 2 Real-time PCR system (Bio-Rad, Hercules, CA, USA) according to the following protocol: 95°C for 3 min, followed by 40 cycles of 95°C for 30 s, 60°C for 30 s, and 72°C for 30 s. The qPCR product was checked in a 2% agarose gel to ensure the specificity of PCR amplification. *E. coli* run-off cells growing in LB medium that have been treated with 300 µg/mL chloramphenicol for over 2 hours was used as the control group (2 hour is enough for DNA replication run-off, *ori/ter* equals 1). Our results showed that the Ct value of *oriC* and *ter* was exactly the same for the run-off cell sample (*ori/ter*=1), demonstrating the amplification efficiency of the *oriC* primers and *ter* primers was exactly the same. Therefore the *ori/ter* ratio is simply equals to 2^ΔCt^ ($\Delta\mathrm{Ct}=\mathrm{Ct}[ter]-\mathrm{Ct}[oriC]$). Calculation of C period is based on $ori/ter= 2^{(C/\tau)}$.

The second method is the DNA increment method as described at Churchward et al(11) and Bipatnath et al(12) by measuring the DNA increment after blocking DNA initiation of exponentially growing *E. coli* cells by addition of 300 µg/mL chloramphenicol or 200 µg/mL rifampicin (run-off experiments). The relative DNA amount change after DNA initiation blockage corresponds to origins (Ori) /genome equivalents for the cell populations. Generally, the results obtained using chloramphenicol and rifampicin were the same as each other. DNA content per cell was obtained through measuring total DNA amount per OD and cell count per OD through plating as described in Basan et al (2015)(5). Therefore, origins/cell can be obtained using genome equivalents per cell times the ori/genome equivalents ratio. D period is further derived based on the equation: $ori/cell= 2^{(C+D)/\tau}$ (9, 13).

**Microscopy and cell size measurement**

The general microscopy method was performed similarly as described in Basan et al(7) with a spinning disc confocal microscope (Zeiss Cell Observer SD, Zeiss, Germany). Cell length (*L*) and width (*W*) of 500-1000 individual cells were extracted using the ImageJ software. The cell volume (*V*) was calculated based on $V=\pi W^{2}/4\cdot(L-\frac{W}{3}).$

**References**

1. Soupene E, van Heeswijk WC, Plumbridge J, Stewart V, Bertenthal D, Lee H, et al. Physiological studies of Escherichia coli strain MG1655: growth defects and apparent cross-regulation of gene expression. J Bacteriol. 2003;185(18):5611-26.

2. You C, Okano H, Hui S, Zhang Z, Kim M, Gunderson CW, et al. Coordination of bacterial proteome with metabolism by cyclic AMP signalling. Nature. 2013;500(7462):301-6.

3. Datsenko KA, Wanner BL. One-step inactivation of chromosomal genes in Escherichia coli K-12 using PCR products. Proc Natl Acad Sci U S A. 2000;97(12):6640-5.

4. Mao XJ, Huo YX, Buck M, Kolb A, Wang YP. Interplay between CRP-cAMP and PII-Ntr systems forms novel regulatory network between carbon metabolism and nitrogen assimilation in Escherichia coli. Nucleic Acids Res. 2007;35(5):1432-40.

5. Dai X, Zhu M, Warren M, Balakrishnan R, Patsalo V, Okano H, et al. Reduction of translating ribosomes enables Escherichia coli to maintain elongation rates during slow growth. Nat Microbiol. 2016;2:16231.

6. Buckstein MH, He J, Rubin H. Characterization of nucleotide pools as a function of physiological state in Escherichia coli. J Bacteriol. 2008;190(2):718-26.

7. Basan M, Zhu M, Dai X, Warren M, Sevin D, Wang YP, et al. Inflating bacterial cells by increased protein synthesis. Mol Syst Biol. 2015;11(10):836.

8. Si F, Li D, Cox SE, Sauls JT, Azizi O, Sou C, et al. Invariance of Initiation Mass and Predictability of Cell Size in Escherichia coli. Curr Biol. 2017;27(9):1278-87.

9. Hill NS, Kadoya R, Chattoraj DK, Levin PA. Cell size and the initiation of DNA replication in bacteria. PLoS Genet. 2012;8(3):e1002549.

10. Waldminghaus T, Weigel C, Skarstad K. Replication fork movement and methylation govern SeqA binding to the Escherichia coli chromosome. Nucleic Acids Res. 2012;40(12):5465-76.

11. Churchward G, Bremer H. Determination of deoxyribonucleic acid replication time in exponentially growing Escherichia coli B/r. J Bacteriol. 1977;130(3):1206-13.

12. Bipatnath M, Dennis PP, Bremer H. Initiation and velocity of chromosome replication in Escherichia coli B/r and K-12. J Bacteriol. 1998;180(2):265-73.

13. Bremer H, Dennis PP. Modulation of chemical composition and other parameters of the cell at different exponential growth rates. Escherichia coli and Salmonella, ed Neidhardt FC (Am Soc Microbiol, Washington,DC), 2nd Ed. 1996:1553–69.
